# Systematic review of the results of fenestrated endovascular aortic repair in octogenarians

**DOI:** 10.1308/rcsann.2023.0032

**Published:** 2023-08-29

**Authors:** M Elahwal, T Richards, A Imsirovic, R Bagga, G Almond, SW Yusuf

**Affiliations:** University Hospitals Sussex NHS Foundation Trust, UK

**Keywords:** FEVAR – Octogenarians – Complex abdominal aortic aneurysm

## Abstract

**Introduction:**

With the increasing life expectancy of Western populations, more octogenarians are presenting with large abdominal aortic aneurysm (AAA). Endovascular repair offers a less invasive alternative and older patients who may not have been offered open repair in the past are now being considered for elective repair with this approach. Age in isolation may not be the only consideration in recommending elective aneurysm repair. We aimed to review the literature on complex endovascular AAA repairs (mainly fenestrated endovascular aortic repair [FEVAR]) in octogenarians.

**Methods:**

A literature search was conducted using the Ovid Medline^®^, Embase^®^ and Cochrane Library databases for articles published up to January 2022. All English language publications from 1995 onwards were eligible for inclusion. Search terms included: “FEVAR”, “F-EVAR”, “fenestrated EVAR”, “fenestrated endovascular aortic repair”, “fenestrated endovascular aneurysm repair”, “fenestrated AAA repair”, “fenestrated endograft”, “fenestrated stent graft”, “fenestrated”, “endograft”, “EVAR”, “octogenarian”, “elderly”, “above 80” and “over 80”.

**Methods:**

The literature search identified 134 potential articles. Following qualitative assessment by two independent appraisers, this was refined to 11 studies, in accordance with the PRISMA (Preferred Reporting Items for Systematic reviews and Meta-Analyses) statement.

**Results:**

The primary outcome measure was 30-day mortality, which was highly variable, ranging from 0% to 9% in octogenarians and from 0% to 5% in non-octogenarians. However, these differences were only found to be statistically significant in two studies. The secondary outcome measures included technical success rates, major adverse events, reintervention rates, freedom from reintervention, target vessel patency, freedom from target branch instability, and length of hospital and intensive care unit stay. No statistically significant differences were found between octogenarians and non-octogenarians. Long-term survival was significantly lower for octogenarians in two studies.

**Conclusions:**

The perioperative outcomes of FEVAR in octogenarians are comparable with those of younger patients. FEVAR therefore appears to be an acceptable option for complex endovascular aneurysm repairs in carefully selected octogenarians. Nevertheless, this review highlights the paucity of published data on the outcomes of endovascular repair of complex aneurysms in octogenarians.

## Introduction

The management and repair of abdominal aortic aneurysms (AAAs) has undergone many significant changes and improvements over the past few decades, especially with the continuous advances in the field of endovascular repair, which allows increasingly complex aneurysms to be treated in individuals who would have previously been considered unsuitable owing to their age and comorbidities.^1^ With the increasing life expectancy of Western populations, more octogenarians are presenting with AAAs. This poses a clinical dilemma as this group of patients is generally considered to be high risk for open complex aneurysm repair and may not have been considered for intervention in the past.^2^

Infrarenal endovascular aortic repair (EVAR) in octogenarians has proved to be safe and effective in this challenging age group, which has encouraged many to undertake complex endovascular repairs, particularly fenestrated and branched endografts, to treat the more complex patients in this cohort.^3^ This trend can be seen in multiple reports from Canada, the US and the UK, where the numbers of elective AAA repairs were noted to have decreased in all age groups except octogenarians (a 70% increase in the Canadian study).^4^ Data from the Nationwide Inpatient Sample database in the US showed that 25% of all EVAR procedures were performed in patients aged >80 years.^5^ Furthermore, data from the National Vascular Registry have shown that 40.6% of fenestrated EVAR (FEVAR)/branched EVAR procedures performed in the UK between 2019 and 2021 were in patients aged 76–85 years, and 3.3% were in those aged 86 years and over.^6^

There is still a degree of heightened caution exercised with patients presenting with complex AAAs in their eighties owing to perceived inferior fitness compared with younger patients, based on previous experience of open aneurysm repairs in this group.^7,8^ This remains a critical deciding factor in offering complex endovascular repairs to these patients.

Another important factor to consider is the long-term results and cost effectiveness of these interventions in the octogenarian cohort. This is particularly true because the rupture risks that were originally quoted appear to be higher than the actual rupture risk, as illustrated by Earnshaw’s review of screening data.^9^ In the current state of intense pressures on various healthcare systems (especially following the COVID-19 pandemic), these factors should not be overlooked.

We aimed to review the literature on the results of complex endovascular AAA repairs in the form of FEVAR in octogenarians in an attempt to ascertain relative mortality and morbidity following these interventions.

## Methods

This review was registered on the PROSPERO database (CRD42022311189). Studies included in the analysis fulfilled the following criteria:

• elective and urgent FEVAR for non-ruptured aneurysms in men or women;
• reported patient demographics (specifically age >80 years);
• reported 30-day mortality rates.

### Search strategy

The literature search was conducted using the Ovid Medline^®^, Embase^®^ and Cochrane Library databases. Although the first reports of complex EVAR were published in 1996 and 1998,^10,11^ all English language publications from 1995 onwards were included. Search terms comprised: ‘FEVAR’, ‘F-EVAR’, ‘fenestrated EVAR’, ‘fenestrated endovascular aortic repair’, ‘fenestrated endovascular aneurysm repair’, ‘fenestrated AAA repair’, ‘fenestrated endograft’, ‘fenestrated stent graft’, ‘fenestrated’, ‘endograft’, ‘EVAR’, ‘octogenarian’, ‘elderly’, ‘above 80’ and ‘over 80’. A search of the Cochrane database was also undertaken using the keywords ‘FEVAR’ and ‘octogenarians’.

Our literature search was undertaken on 15 January 2022. The full search strategy is outlined in Table 1.

**Table 1:** Full search strategy

**Information sources** MEDLINE and EMBASE search conducted through the Ovid database
**Search terms** EMBASE <1995 to 2022 Week 02> Ovid MEDLINE(R) ALL <1995 to January 15, 2022>
1. FEVAR.mp. [mp=ti, ab, hw, tn, ot, dm, mf, dv, kf, fx, dq, nm, ox, px, rx, ui, sy] 709 2. F-EVAR.mp. [mp=ti, ab, hw, tn, ot, dm, mf, dv, kf, fx, dq, nm, ox, px, rx, ui, sy] 163 3. Fenestrated EVAR.mp. [mp=ti, ab, hw, tn, ot, dm, mf, dv, kf, fx, dq, nm, ox, px, rx, ui, sy] 251 4. Fenestrated endovascular aortic repair.mp. [mp=ti, ab, hw, tn, ot, dm, mf, dv, kf, fx, dq, nm, ox, px, rx, ui, sy] 200 5. Fenestrated endovascular aneurysm repair.mp. [mp=ti, ab, hw, tn, ot, dm, mf, dv, kf, fx, dq, nm, ox, px, rx, ui, sy] 496 6. Fenestrated AAA repair.mp. [mp=ti, ab, hw, tn, ot, dm, mf, dv, kf, fx, dq, nm, ox, px, rx, ui, sy] 0 7. Fenestrated endograft.mp. [mp=ti, ab, hw, tn, ot, dm, mf, dv, kf, fx, dq, nm, ox, px, rx, ui, sy] 350 8. Fenestrated Stent Graft.mp. [mp=ti, ab, hw, tn, ot, dm, mf, dv, kf, fx, dq, nm, ox, px, rx, ui, sy] 509 9. Fenestrated.mp. [mp=ti, ab, hw, tn, ot, dm, mf, dv, kf, fx, dq, nm, ox, px, rx, ui, sy] 10,626 10. Endograft.mp. [mp=ti, ab, hw, tn, ot, dm, mf, dv, kf, fx, dq, nm, ox, px, rx, ui, sy] 7,520 11. EVAR.mp. [mp=ti, ab, hw, tn, ot, dm, mf, dv, kf, fx, dq, nm, ox, px, rx, ui, sy] 12,209 12. 9 and 10 924 13. 9 and 11 1,175 14. Octogenarian.mp. [mp=ti, ab, hw, tn, ot, dm, mf, dv, kf, fx, dq, nm, ox, px, rx, ui, sy] 4,144 15. Elderly.mp. [mp=ti, ab, hw, tn, ot, dm, mf, dv, kf, fx, dq, nm, ox, px, rx, ui, sy] 913,100 16. Above 80.mp. [mp=ti, ab, hw, tn, ot, dm, mf, dv, kf, fx, dq, nm, ox, px, rx, ui, sy] 6,612 17. Over 80.mp. [mp=ti, ab, hw, tn, ot, dm, mf, dv, kf, fx, dq, nm, ox, px, rx, ui, sy] 31,633 18. 1 or 2 or 3 or 4 or 5 or 6 or 7 or 8 or 12 or 13 2,598 19. 14 or 15 or 16 or 17 948,177 20. 18 and 19 130 21. remove duplicates from 20 120

Studies describing juxta/pararenal AAAs as well as thoracoabdominal aortic aneurysms (TAAAs) and FEVAR performed for salvage of type Ia endoleaks were included in the review. Data for cohorts treated with both branched endografts and fenestrated endografts were used if it was not possible to extract FEVAR data separately. Devices from all manufacturers were included. Case reports, conference abstracts and case series with fewer than five patients were excluded. Studies involving parallel grafts were not included in the review unless it was possible to eliminate these patients from the analysis.

### Selection process

The search was conducted independently by two researchers (ME and TR), with disputes settled by SWY. A data collection tool was created using Microsoft Excel^®^, and data entry for the studies included in the review was undertaken by ME and verified by TR.

### Outcome measures

The primary outcome measure studied in this review was 30-day mortality. Major adverse events, rates of reintervention, freedom from reintervention, freedom from branch instability, long-term survival rates and length of stay were recorded as secondary outcome measures.

### Risk of bias assessment

The Newcastle–Ottawa quality assessment scales for cohort and case controlled studies were employed to evaluate the quality of the included studies.^12^ This was assessed independently by two reviewers (ME and TR).

## Results

**Figure 1:**
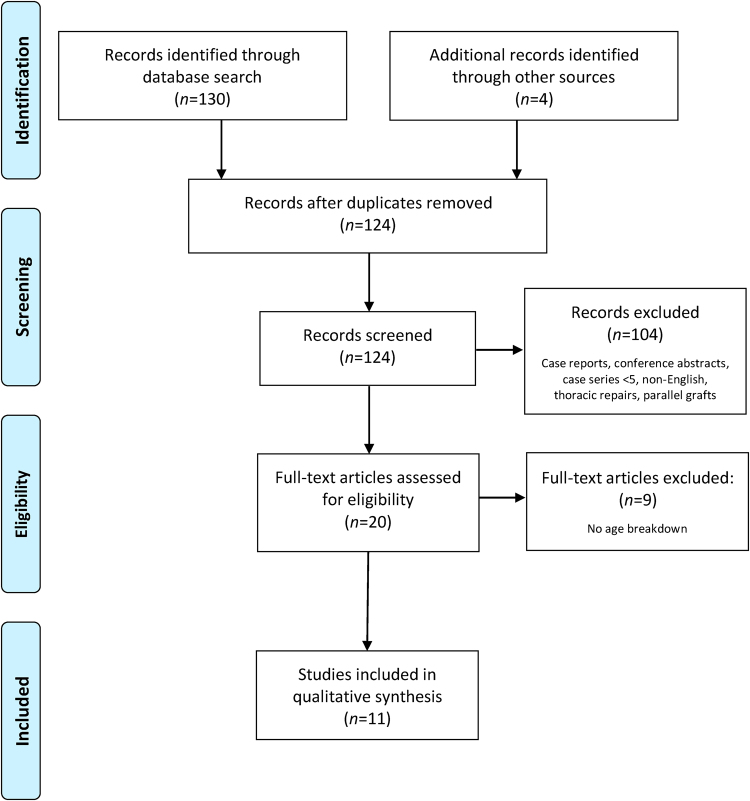
Flowchart of study selection

A total of 130 records were identified from the Ovid Medline^®^ and Embase^®^ databases; the Cochrane Library database search resulted in zero matches. A further four records were found from other sources (Google Scholar™ search). Following removal of duplicates, 124 records were screened. Of these, 104 were excluded based on their titles and abstracts, and 20 full-text articles were reviewed. Another nine articles were subsequently excluded as they did not include a breakdown of age, meaning eleven studies were included in the qualitative synthesis (Figure 1).^1,2,13–21^

### Study characteristics

The characteristics of the 11 included studies are summarised in Table 2. Six papers described single centre cohorts,^14,16–20^ two studies included data from two centres^1,13^ and three studies involved national level cohorts.^2,15,21^ Data in most of the studies were collected prospectively in databases for complex AAA repairs and were studied retrospectively.^1,2,13,17–19,21^ The risk of selection bias in all studies is recognised and has been addressed in multiple studies.^1,2,13,16^

**Table 2:** Characteristics of the 11 included studies

Study	Country	Study design	Study period	Number of centres	Urgency	Total patients
Henstra, 2020^1^	Netherlands	Retrospective cohort	2001–2016	2	Elective non-ruptured*	272
Hertault, 2014^13^	France, Sweden	Prospective cohort	2002–2016	2	Elective non-ruptured	288
Lisy, 2019^14^	Germany	Retrospective case series	2015–2016	1	Elective non-ruptured	9
Locham, 2018^15^	US	Retrospective cohort	2006–2015	National	Elective non-ruptured	242
Makaloski, 2018^16^	Germany	Retrospective cohort	2015–2017	1	Emergency ruptured, elective non-ruptured	207
Motta, 2021^2^	US	Prospective cohort	2012–2018	National	Elective, urgent non-ruptured	893
Pini, 2020^17^	Italy	Retrospective cohort	2010–2019	1	Elective non-ruptured	243
Rolls, 2014^18^	UK	Retrospective case series	2011–2012	1	Elective non-ruptured	13
Timaran, 2017^19^	US	Retrospective cohort	33 months	1	Elective non-ruptured	85
Tran, 2019^20^	US	Retrospective cohort	2009–2015	1	Elective non-ruptured	103 (17 FEVAR)
Zil-E-Ali, 2022^21^	US, Canada	Retrospective cohort	2010–2019	National	Emergency ruptured, elective non-ruptured	5,507
FEVAR = fenestrated endovascular aortic repair *Includes one patient with contained rupture, whose custom-made graft was available on shelf at presentation.						

Two studies included only octogenarians^15,20^ while the remaining nine included a comparative group of non-octogenarians. A total of 7,776 patients were identified, of whom 1,772 were octogenarians. Table 3 outlines the number of octogenarian and non-octogenarian patients in each study as well as the average age of the two groups.

**Table 3:** Average age of octogenarians and non-octogenarians

Study	Octogenarians		Non-octogenarians	
	Number of patients	Average age in years (range)	Number of patients	Average age in years (range)
Henstra, 2020^1^	42	82.3±2.5	230	71.4±6.1
Hertault, 2014^13^	33	82.3 (81.2–83.3)	255	70.3 (69.6–71.1)
Lisy, 2019^14^	2	84±4	7	73
Locham, 2018^15^	242	83	0	–
Makaloski, 2018^16^	38 (27 elective, 11 emergency)	82 (81–84)	169 (121 elective, 48 emergency)	72 (65–76)
Motta, 2021^2^	195	Not reported	698	Not reported
Pini, 2020^17^	35	82±2	208	72±5
Rolls, 2014^18^	5	85.4	8	71.2
Timaran, 2017^19^	18	84 (81–86)	67	72 (67–76)
Tran, 2019^20^	103 (17 FEVAR)	84.5 (80–95)	0	–
Zil-E-Ali, 2022^21^	1,145 (924 elective, 213 emergency)	83.83±2.98	4,362 (3,429 elective, 879 emergency)	66.31±11.61
FEVAR = fenestrated endovascular aortic repair				

Descriptors of the AAAs and details on the types of repair performed in each of the studies are provided in Table 4. All studies included elective repairs, with only two studies incorporating ruptured AAA and the use of physician modified endografts for their repair.^16,21^ In one study, a single patient with a contained rupture was included owing to their custom-made device being on shelf and used in an urgent fashion.^1^ It was, however, possible to extract data pertaining to 30-day mortality in cases of non-rupture repairs in all studies but one.^21^ This was despite our attempts to contact the corresponding authors of all studies to request the missing data via their correspondence email addresses.

**Table 4:** AAA descriptors for the included studies

Study	Mean AAA diameter in mm (range)		AAA extents included	AAA extent details		Repair type	Grafts used in repair	Comparator repair
	Octogenarians	Non-octogenarians		Octogenarians	Non-octogenarians			
Henstra, 2020^1^	65.3±8.4	63.3±8.6	JR, PR, Ia	48% short neck IR 52% JR 7% Ia	40% short neck IR 55% JR 6% SR 5% Ia	FEVAR	Zenith^®^, Anaconda™	None
Hertault, 2014^13^	63.1 (59.4–66.9)	58.7 (57.4–59.9)	JR, PR	–	–	FEVAR	Zenith^®^	None
Lisy, 2019^14^	66.5	56.1	JR, Ia	100% JR	100% JR	FEVAR	Anaconda™	None
Locham, 2018^15^	–	–	–	–	–	FEVAR	–	Open repair in octogenarians
Makaloski, 2018^16^	58 (54–69)	62 (54–73)	JR, Ia, TAAA	53% JR 48% TAAA	41% JR 55% TAAA	FEVAR/BEVAR	–	None
Motta, 2021^2^	67±14	65±12	JR, PR, TAAA	34% Type I–III, V TAAA 66% JR, type IV TAAA	39% Type I–III, V TAAA 61% JR, type IV TAAA	FEVAR/BEVAR	Zenith^®^	None
Pini, 2020^17^	64±12	60±10	JR, PR, TAAA	38% TAAA	14% TAAA	FEVAR/BEVAR	Zenith^®^	None
Rolls, 2014^18^	76.2	70.5	JR, TAAA	80% JR 20% Type IV TAAA	62.5% JR 37.5% Type IV TAAA	FEVAR	Anaconda™	None
Timaran, 2017^19^	57 (55–64)	55 (53–62)	JR, Ia, TAAA	39% JR 55% SR 5% Type IV TAAA	18% JR 67% SR 15% Type IV TAAA	FEVAR	Zenith^®^	None
Tran, 2019^20^	IR: 58.3±13.5 Complex: 64.4±12.8	–	JR, PR, TAAA	66% IR 34% “complex”	–	FEVAR/chimney EVAR*	Zenith^®^	IR EVAR in octogenarians
Zil-E-Ali, 2022^21^	70.5% >55mm	78.5% >55mm	JR	–	–	FEVAR	–	None
AAA = abdominal aortic aneurysm; BEVAR = branched endovascular aortic repair; EVAR = endovascular aortic repair; FEVAR = fenestrated endovascular aortic repair; IR = infrarenal; JR = juxtarenal; PR = pararenal; SR = suprarenal; TAAA = thoracoabdominal aortic aneurysm; Ia = type Ia endoleak								
*Only FEVAR data (17 patients) were extracted and analysed in our review.								

Ten studies included patients with juxtarenal/pararenal AAA,^1,2,13,14,16–21^ with four studies including patients with type Ia endoleaks^1,14,16,19^ and six studies including patients with TAAA.^2,16–20^ One study did not specify the anatomical extent of the treated aneurysms.^15^

Three studies included patients treated with branched endografts as well as fenestrated endografts but results stratified according to each modality were not outlined in the manuscripts.^2,16,17^ Although we attempted to contact the corresponding authors of these manuscripts via their correspondence email addresses to obtain data pertaining to fenestrated endografts separately, we were unsuccessful except in one case.

One study included patients treated with parallel grafts (chimney EVAR).^20^ Nevertheless, it was possible to exclude these patients and analyse only patients receiving fenestrated repairs.

Three studies did not specify the type of endograft used.^15,16,21^ Six studies included devices from Cook Medical (Bloomington, IN, US)^1,2,13,17,19,20^ and three studies included devices from Terumo Aortic (Inchinnan, UK).^1,14,18^

### Risk of bias assessment

The Newcastle–Ottawa quality assessment scales for cohort and case controlled studies were employed to evaluate the quality of the included studies.^12^ Six studies were found to be of good quality (although risk of selection bias was acknowledged by the authors of four of these) and five studies were found to have a high risk of bias (Table 5).

**Table 5:** Risk of bias assessment using the Newcastle–Ottawa quality assessment scales for cohort and case controlled studies^12^

	Henstra, 2020^1^^a^	Hertault, 2014^13^^b^	Lisy, 2019^14^^c^	Locham, 2018^15^^a^	Makaloski, 2018^16^^a^	Motta, 2021^2^^b^	Pini, 2020^17^^a^	Rolls, 2014^18^^c^	Timaran, 2017^19^^a^	Tran, 2019^20^^a^	Zil-E-Ali, 2022^21^^a^
Representativeness of the exposed cohort	*	*	–	*	*	*	–	–	*	*	*
Selection of the non-exposed cohort	–	–	–	–	–	–	–	–	–	–	–
Ascertainment of exposure	*	*	*	*	*	*	*	*	*	*	*
Demonstration that outcome of interest was not present at start of study	*	*	*	*	*	*	*	*	*	*	*
Comparability of cohorts on the basis of design or analysis	**	**	–	–	**	**	**	–	**	–	–
Assessment of outcome	*	*	*	*	*	*	*	*	*	*	*
Was follow-up long enough for outcomes to occur	*	*	*	*	*	*	*	*	*	*	*
Adequacy of follow-up of cohorts	*	*	*	*	*	*	*	*	*	*	*
**Score**	**8 (good)**	**8 (good)**	**5 (poor)**	**6 (poor)**	**8 (good)**	**8 (good)**	**7 (good)**	**5 (poor)**	**8 (good)**	**6 (poor)**	**6 (poor)**
^a^Retrospective cohort; ^b^Prospective cohort; ^c^Retrospective case series											

### Patient populations

Table 6 shows the baseline characteristics of the patients in the included studies. A significantly lower baseline estimated glomerular filtration rate in octogenarians than in non-octogenarians was observed in four studies^1,14–16^ and a significantly higher incidence of hypercholesterolaemia was noted by Henstra *et al*.^1^ A significantly higher incidence of cancer as well as significantly lower incidences of chronic obstructive pulmonary disease and hypercholesterolaemia in octogenarians were reported by Motta *et al*.^2^ Otherwise, both groups of patients were similar in terms of baseline characteristics.

**Table 6:** Baseline characteristics of patients in octogenarians and non-octogenarians

	Diabetes	Hypertension	Hypercholesterolaemia	IHD	COPD	Renal function	Smoking
Henstra, 2020^1^						*eGFR*	
Octogenarians	3 (7%)	28 (67%)	21 (50%)	29 (69%)	10 (24%)	61.4±17.4	–
Non-octogenarians	37 (16%)	183 (80%)	159 (69%)	137 (60%)	82 (36%)	74.5±22.1	–
*p*-value	*p*=0.14	*p*=0.98	***p*=0.01**	*p*=0.24	*p*=0.15	***p*<0.01**	–
Hertault, 2014^13^						*Renal insufficiency*	
Octogenarians	5 (15.6%)	26 (78.8%)	–	13 (40.6%)	10 (31.2%)	4 (16.0%)	9 (27.3%)
Non-octogenarians	43 (17.6%)	172 (69.9%)	–	121 (48.6%)	93 (38.6%)	35 (17.1%)	136 (55.3%)
*p*-value	*p*=0.787	*p*=0.292	–	*p*=0.396	*p*=0.893	*p*=0.622	–
Lisy, 2019^14^							
Octogenarians	0 (0%)	1 (50%)	2 (100%)	1 (50%)	1 (50%)	–	1 (50%)
Non-octogenarians	1 (14.3%)	7 (100%)	6 (85.7%)	3 (42.8%)	2 (28.6%)	–	3 (42.8%)
Locham, 2018^15^						*Creatinine*	
	31 (12.8%)	199 (82.2%)	–	–	44 (18.2%)	1.1 (0.9–1.4)	37 (15.3%)
Makaloski, 2018^16^						*Creatinine*	
Octogenarians	22 (13%)	131 (78%)	38 (23%)	79 (47%)	37 (22%)	1.1 (0.9–1.3)	60 (36%)
Non-octogenarians	3 (8%)	30 (79%)	11 (29%)	18 (47%)	10 (26%)	1.2 (1.0–1.6)	9 (24%)
*p*-value	*p*=0.58	*p*=1	*p*=0.4	*p*=1	*p*=0.53	***p*=0.04**	*p*=0.19
Motta, 2021^2^						*ESRD*	
Octogenarians	59 (30%)	177 (91%)	127 (65%)	91 (47%)	65 (33%)	1 (0.5%)	–
Non-octogenarians	204 (29%)	632 (91%)	528 (76%)	350 (50%)	290 (42%)	9 (1.3%)	–
*p*-value	*p*=0.78	*p*=0.92	***p*=0.003**	*p*=0.39	***p*=0.038**	*p*=0.7	–
Pini, 2020^17^						*CKD*	
Octogenarians	7 (20%)	31 (88%)	21 (60%)	23 (65%)	18 (47%)	15 (43%)	7 (20%)
Non-octogenarians	22 (11%)	195 (94%)	151 (73%)	65 (31%)	87 (42%)	85 (40%)	75 (36%)
*p*-value	*p*=0.21	*p*=0.67	*p*=0.58	*p*=0.48	*p*=0.83	*p*=0.35	*p*=0.43
Rolls, 2014^18^							
Octogenarians	1 (20%)	5 (100%)	0 (0%)	1 (20%)	0 (0%)	–	3 (60%)
Non-octogenarians	2 (25%)	8 (100%)	3 (37.5%)	3 (37.5%)	3 (37.5%)	–	6 (75%)
Timaran, 2017^19^						*Creatinine*	
Octogenarians	22%	67%	55%	61%	45%	1.07 (0.94–1.47)	55%
Non-octogenarians	18%	83%	74%	52%	39%	1.14 (0.92–1.35)	55%
*p*-value	*p*=0.7	*p*=0.1	*p*=0.1	*p*=0.5	*p*=0.7	*p*=0.6	*p*=1
Tran, 2019^20^*						*CKD*	
	4 (11.4%)	30 (85.7%)	31 (88.5%)	22 (62.8%)	17 (48.6%)	25 (71.4%)	28 (80%)
Zil-E-Ali, 2022^21^						*Pre-op dialysis*	
Octogenarians	193 (16.4%)	995 (87.4%)	–	296 (26%)	298 (26.2%)	19 (1.7%)	812 (71.4%)
Non-octogenarians	824 (18.2%)	3,666 (84.7%)	–	1,084 (25.1%)	1,404 (32.5%)	85 (2%)	3,617 (83.8%)
*p*-value	*p*=0.101	***p*=0.020**	–	*p*=0.513	***p*<0.001**	*p*=0.722	***p*<0.001**
CKD = chronic kidney disease; COPD = chronic obstructive pulmonary disease; eGFR = estimated glomerular filtration rate; ESRD = end-stage renal disease; IHD = ischaemic heart disease							
*Reported baseline characteristics for “complex repairs”, which included fenestrated and chimney endovascular aneurysm repairs without distinction							

### Primary outcome

The primary outcome measure studied in this review was 30-day mortality. This was highly variable, ranging from 0% to 9% in octogenarian cohorts and from 0% to 5% in non-octogenarian cohorts (Table 7). However, these differences were only found to be statistically significant in the studies by Hertault *et al*^13^ and Zil-E-Ali *et al*.^21^ The pooled mortality rate for elective repairs in octogenarians was 5.9% (104 patients) while that for elective repairs in non-octogenarians was 4.2% (247 patients). The pooled mortality was found to be significantly higher in octogenarians using the chi-squared test (*p*=0.002).

**Table 7:** Thirty-day mortality rates in octogenarians and non-octogenarians

	Mortality		*p*-value
	Octogenarians	Non-octogenarians	
Henstra, 2020^1^	1/42 (2.4%)	7/230 (3.0%)	0.051
Hertault, 2014^13^	3/33 (9.1%)	4/255 (1.6%)	**0.004**
Lisy, 2019^14^	0/2 (0%)	0/7 (0%)	–
Locham, 2018^15^	10/242 (4.1%)	–	–
Makaloski, 2018^16^	–	–	–
Elective	2/27 (7.4%)	4/121 (3.3%)	0.354
Urgent	5/11 (45.5%)	8/48 (16.7%)	0.119
Patients undergoing FEVAR	2/27 (7.4%)	2/86 (2.3%)	0.212
Motta, 2021^2^	1/195 (0.5%)	9/698 (1.3%)	0.366
Pini, 2020^17^	2/35 (7%)	7/208 (3.5%)	0.515
Rolls, 2014^18^	0/5 (0%)	0/8 (0%)	–
Timaran, 2017^19^	0/18 (0%)	0/67 (0%)	–
Tran, 2019^20^	1/17 (5.9%)	–	–
Zil-E-Ali, 2022^21^	84/1,145 (7.3%)	219/4,362 (5.0%)	**<0.001**
**Total**	**104/1,761 (5.9%)**	**247/5,921 (4.2%)**	**0.002**
FEVAR = fenestrated endovascular aortic repair			

### Secondary outcomes

Technical success rate was reported in nine studies and ranged from 78% to 100%.^1,2,13,14,16–19^ There was no significant difference between technical success in octogenarians and non-octogenarians in any of the studies.

The definition of major adverse events was inconsistent throughout the studies included in this review. Nevertheless, the Society for Vascular Surgery reporting standards were cited in three studies.^2,13,21^ The most commonly reported adverse events across all studies were myocardial infarctions, spinal cord ischaemia, acute kidney injury, renal failure with haemodialysis, stroke, respiratory complications/failure, bowel ischaemia and acute limb ischaemia.

Table 8 shows the different complication rates reported in the various studies. No significant differences in major adverse events were found between octogenarians and non-octogenarians.

**Table 8:** Major adverse events and their details in octogenarians and non-octogenarians

	Major adverse events	MI	SCI	AKI	RF with HD	Stroke	Respiratory
Henstra, 2020^1^							
Octogenarians	–	0 (0%)	0 (0%)	–	0 (0%)	0 (0%)	0 (0%)
Non-octogenarians	–	–	5 (2%)	–	5 (2%)	–	–
*p*-value	–	–	*p*=0.34	–	*p*=0.34	–	–
Hertault, 2014^13^^b^	6 (18%)	–	–	1 (3%)	2 (6%)	2 (6%)	1 (3%)
Lisy, 2019^14^							
Octogenarians	–	0 (0%)	0 (0%)	–	0 (0%)	0 (0%)	0 (0%)
Non-octogenarians	–	–	–	–	–	–	–
Locham, 2018^15^	–	14 (5.8%)^a^	–	5 (2.1%)	1 (0.4%)	1 (0.4%)	14 (5.8%)^a^
Makaloski, 2018^16^							
Octogenarians	–	–	6 (16%)	10 (26%)	1 (3%)	1 (3%)	3 (8%)
Non-octogenarians	–	–	27 (16%)	23 (14%)	4 (2%)	5 (3%)	13 (8%)
*p*-value	–	–	*p*=0.89	*p*=0.21	–	*p*=1	*p*=1
Motta, 2021^2^							
Octogenarians	18 (9.2%)	–	–	–	–	–	–
Non-octogenarians	68 (9.7%)	–	–	–	–	–	–
*p*-value	*p*=0.83	–	–	–	–	–	–
Pini, 2020^17^	–	–	–	–	–	–	–
Rolls, 2014^18^							
Octogenarians	–	–	–	0 (0%)	–	–	–
Non-octogenarians	–	–	–	1 (12.5%)	–	–	–
Timaran, 2017^19^							
Octogenarians	34%	5%	–	1 (7.6%)	–	–	0%
Non-octogenarians	–	6%	–	–	–	–	5%
*p*-value	–	*p*>0.5	–	–	–	–	*p*>0.5
Tran, 2019^20^^b^	23.5%	–	–	–	–	–	–
Zil-E-Ali, 2022^21^							
Octogenarians	–	29 (2.6%)	8 (1.1%)	31 (7.5%)	6 (1.5%)	29 (2.6%)	–
Non-octogenarians	–	109 (2.5%)	46 (1.8%)	128 (6.9%)	60 (3.2%)	119 (2.8%)	–
*p*-value	–	*p*=0.955	*p*=0.247	*p*=0.265	–	*p*=0.701	–
AKI = acute kidney injury; HD = haemodialysis; MI = myocardial infarction; RF = renal failure; SCI = spinal cord ischaemia							
^a^Cardiopulmonary complications reported together; ^b^Results reported for octogenarian group only							

Reintervention rates^1,14,15,19,21^ and freedom from reintervention^1,2,17,19,21^ were each reported in five studies, as were target vessel patency and freedom from target branch instability.^1,2,13,18,19^ No significant differences were reported between octogenarians and non-octogenarians in terms of these outcomes except for a higher rate of freedom from reintervention for octogenarians in one study (Table 9).^19^

**Table 9:** Rates of reintervention, freedom from reintervention and freedom from branch instability, long-term survival rates and length of stay

	Reintervention	Freedom from reintervention	Freedom from target vessel instability	Long-term survival	Length of hospital stay in days (range)	Length of ICU stay in days (range)
Henstra, 2020^1^						
Octogenarians	3 (7%)	–	85 (96.6%)	–	5.8±3.4 (1–18)	0.2±0.6 (0–3)
Non-octogenarians	20 (8.7%)	–	508 (94.6%)	–	6.9±10.8 (1–120)	0.9±4.6 (0–62)
*p*-value	–	*p*=0.95	*p*=0.56	*p*=0.08	*p*=0.54	*p*=0.39
Hertault, 2014^13^						
Octogenarians	7 (21.2%)*	–	31 (93.9%)*	77.8%	8	4 (1–18)
Non-octogenarians	–	–	–	89%	7	5 (0–67)
*p*-value	–	–	–	–	*p*=0.66	*p*=0.337
Lisy, 2019^14^	–	–	–	–	–	–
Locham, 2018^15^	–	–	–	–	3 (2–5)*	–
Makaloski, 2018^16^						
Octogenarians	10 (26%)	–	–	–	14 (9–19)	4 (2–6)
Non-octogenarians	31 (18%)	–	–	–	13 (8–22)	4 (3–7)
*p*-value	*p*=0.23	–	–	–	*p*=0.62	*p*=0.35
Motta, 2021^2^						
Octogenarians	–	60.3%	77.5%	29%	8.2±27	–
Non-octogenarians	–	59.5%	75.9%	66.2%	5.7±6.3	–
*p*-value	–	*p*=0.73	*p*=0.57	***p*<0.01**	*p*=0.19	–
Pini, 2020^17^						
Octogenarians	–	85±3%	–	62±10%	–	–
Non-octogenarians	–	–	–	82±3%	–	–
*p*-value	–	–	–	***p*=0.003**	–	–
Rolls, 2014^18^						
Octogenarians	–	–	100%	–	7.8 (5–12)	–
Non-octogenarians	–	–	97%	–	8.5 (4–20)	–
Timaran, 2017^19^						
Octogenarians	1 (7.6%)	95%	100%	75%	3.5 (2–5)	3 (2–5)
Non-octogenarians	12 (17.9%)	70%	61 (91%)	91%	4 (2–5)	2 (1–3)
*p*-value	*p*=0.09	***p*=0.04**	–	*p*=0.1	*p*=0.87	*p*=0.76
Tran, 2019^20^	–	–	–	52.7%	6±4.5*	–
Zil-E-Ali, 2022^21^						
Octogenarians	40 (5.6%)	97%	–	58.25%	6.26±13	2.83±5.01
Non-octogenarians	143 (6%)	98%	–	64.6%	8.22±35.46	3.32±6.11
*p*-value	*p*=0.699	*p*=0.433	–	***p*<0.05**	*p*=0.326	***p*<0.001**
ICU = intensive care unit						
*Only reported in octogenarians						

Long-term survival was reported in seven studies.^1,2,13,17,19–21^ Octogenarians exhibited significantly lower long-term survival in two studies^17,21^ and a significantly lower long-term survival only when studied after more than three years in another.^2^ The results for long-term survival are summarised in Table 9.

Length of hospital stay was reported in eight studies^1,2,13,16,18–21^ and length of intensive care unit (ICU) stay was reported in five studies.^1,13,16,19,21^ There were no significant differences between octogenarians and non-octogenarians in length of stay except for a shorter length of ICU stay in octogenarians in one study (Table 9).^21^

## Discussion

This review highlights the paucity and relative low quality of data on the results of complex EVAR in octogenarians. Many of the studies found were single centre studies with small numbers of patients in case series or retrospective cohorts and no level I evidence in the form of randomised controlled trials although it is understandable that it would be difficult to organise a randomised controlled trial. It may, however, be possible to collect more data on the natural history of aneurysms in the 5.5–6.0cm range by offering a “watchful waiting” approach to patients in this age group.^9^

The findings of our review show that the perioperative outcomes of complex endovascular repairs in octogenarians are comparable with those in non-octogenarians, with no statistically significant differences in terms of 30-day mortality or morbidities except in two studies.^13,21^ Hertault *et al* indicated that all octogenarian deaths in their cohort occurred in patients who had required early reinterventions and consequently advocated that pushing the technical limits of difficult anatomy should be avoided in octogenarians.^13^ In the study by Zil-E-Ali *et al*, 20% of the patients were treated in an emergency fashion, which might be a factor contributing to the higher mortality rate in octogenarians in their cohort.^21^

The counterargument remains that the octogenarian patients considered for FEVAR were similar in fitness and baseline characteristics to their younger counterparts, and were therefore not indicative of the general fitness of the whole octogenarian population. This concern over selection bias is still valid. It is likely that the data in these studies may only be applicable to carefully selected patients who are physiologically robust and have a good life expectancy.^1,2,13,16^

We also acknowledge the possible publication bias due to results of complex endovascular AAA repairs being reported by highly specialised and experienced centres, which may again not be generalisable. Centralisation of vascular services in the UK with high volume centres providing complex aneurysm surgery does, however, make these data relevant to UK patients. The 2022 National Vascular Registry report shows that the in-hospital mortality rate for complex FEVAR between January 2019 and December 2021 was 2.6% (95% confidence interval: 1.7–3.7%), with 44.7% of patients aged >75 years.^6^

The pooled mortality rates from our included studies (5.9% in octogenarians and 4.2% in non-octogenarians) remain in line with the previously reported mortality rates in the literature for elective FEVAR, ranging between 2% and 5%.^22,23^ Furthermore, when compared with mortality results for open surgical repair of short neck infrarenal and juxtarenal AAA in octogenarians, which range from 4%^24^ to 8.5%^15^ and even up to 20%,^8^ the mortality rates following FEVAR in our review seem to confirm the viability of this treatment option in octogenarians.

There were no significant differences between octogenarians and non-octogenarians for technical success, freedom from target vessel instability or freedom from reinterventions. The length of hospital stay and length of ICU stay were also similar.

It was not always possible to extract meaningful data to compare the different extents of aortic aneurysm repairs (TAAA vs juxtarenal/pararenal aneurysms) as the inclusion criteria and grouping methods were inconsistent. We do accept that TAAA repairs carry inherently higher postoperative morbidity and mortality risks, and this should be independently studied in the future to assess the results in this challenging age group. This would no doubt have an effect on the reported results of the papers including a broad range of aneurysm extents. The results of FEVAR specifically in juxtarenal aneurysms are currently being evaluated in the UK-COMPASS trial and more data on octogenarians included in this study will further enrich our understanding.^25^

With regard to complex repairs of ruptured aneurysms, we elected to exclude these patients from our analysis as the pathophysiology and outcomes are very diverse, and present a significant challenge on their own. We believe this should be addressed on a case-by-case basis until there are more robust data in the literature.

The long-term survival rates of octogenarians remained comparable with those of younger patients for at least three years, with one study reporting a lower survival rate in octogenarians further down the line.^2^ This could be expected given the naturally shorter life expectancy of octogenarians compared with younger members of the population.

### Study limitations

Our review was limited in terms of the small number of studies, the diverse inclusion/exclusion criteria, aneurysm extents and techniques used in all the studies. The aim was to extract the most relevant information from studies where this was possible but the difficulty in obtaining patient level data from most of these studies (despite attempts to contact the corresponding authors for these data) did not allow us to perform a meta-analysis of the results.

## Conclusions

This review highlights that FEVAR remains an acceptable option for complex EVARs in selected octogenarians, with morbidity and mortality rates similar to those generally expected in all patients. However, these results are probably only applicable to carefully selected patients without significant comorbidities and with a good fitness level.

There is a paucity of data on the outcomes of endovascular repair of complex aneurysms in octogenarians. National registries are likely to fill this gap and more collaboration is required to collate these data for reliable analysis in the future, in particular with regard to outcomes beyond 30 days in order to provide a better understanding of the overall impact on survival, quality of life and cost effectiveness.
